# Distinct trajectories of symptomatic response in ulcerative colitis during filgotinib therapy: A post hoc analysis from the SELECTION study

**DOI:** 10.1002/ueg2.12686

**Published:** 2024-10-25

**Authors:** Stefan Schreiber, Brian G. Feagan, Edouard Louis, Tadakazu Hisamatsu, Toshifumi Hibi, Louis Dron, Corinne Jamoul, Haridarshan Patel, Kristina Harris, Virginia Taliadouros, Alessandra Oortwijn, Laurent Peyrin‐Biroulet

**Affiliations:** ^1^ Department of Medicine I University Hospital Schleswig‐Holstein Kiel Germany; ^2^ Alimentiv, Inc. London Ontario Canada; ^3^ Division of Gastroenterology Department of Medicine Western University London Ontario Canada; ^4^ Department of Gastroenterology University Hospital of Liège Liège Belgium; ^5^ Department of Gastroenterology and Hepatology Kyorin University School of Medicine Tokyo Japan; ^6^ Center for Advanced IBD Research and Treatment Kitasato University Kitasato Institute Hospital Tokyo Japan; ^7^ Cytel Vancouver British Columbia Canada; ^8^ Galapagos NV Mechelen Belgium; ^9^ Galapagos S.r.l Milan Italy; ^10^ Galapagos B.V Leiden Netherlands; ^11^ Department of Gastroenterology Nancy University Hospital Nancy France; ^12^ INSERM NGERE University of Lorraine Nancy France; ^13^ INFINY Institute Nancy University Hospital Nancy France; ^14^ FHU‐CURE Nancy University Hospital Nancy France; ^15^ Groupe Hospitalier Privé Ambroise Paré ‐ Hartmann Paris IBD Center Paris France; ^16^ Division of Gastroenterology and Hepatology McGill University Health Centre Montreal Quebec Canada

**Keywords:** clinical trials, comprehensive disease control, filgotinib, inflammatory bowel disease, Janus kinase 1 inhibitor, symptom trajectories, ulcerative colitis

## Abstract

**Background:**

Filgotinib is an oral, once‐daily, Janus kinase 1 preferential inhibitor approved for treatment of ulcerative colitis (UC) following the phase 2b/3 SELECTION trial. Identification of patient populations and factors associated with long‐term treatment response trajectories may improve UC management.

**Objective:**

We aimed to identify and describe distinct patient subgroups of response to filgotinib based on partial Mayo Clinic Score (pMCS) trajectories over time.

**Methods:**

In these *post hoc* analyses of SELECTION, group‐based trajectory modeling (GBTM) was applied to pMCS to describe groups of distinct, symptom‐based patient trajectories using data from patients who responded to filgotinib 200 or 100 mg and continued receiving filgotinib up to week 58. Patient demographics, disease characteristics, and week 10 response were compared between the groups. Achievement of a patient‐level multi‐component endpoint of comprehensive disease control (CDC) was assessed in each group.

**Results:**

GBTM identified five distinct patient populations with different response trajectories; 67.5% of patients had beneficial trajectories. The beneficial trajectory groups generally had higher proportions of patients who were recently diagnosed (<1 year), were receiving filgotinib 200 mg and were biologic‐naive versus the relapsing trajectory groups (4%–9% vs. 4%–5%; 43%–65% vs. 36%–46%; 54%–70% vs. 35%–58%, respectively). Furthermore, 55.4% of patients had sustained beneficial trajectories, with low baseline endoscopic subscores (≥43% of patients had a subscore of 2) and strong week 10 FCP responses (≥61% of patients with >50% decrease in FCP from baseline). Sustained beneficial trajectory groups had a higher probability of achieving CDC at week 58 than other groups (31%–32% vs. 0%–7%).

**Conclusions:**

Beneficial long‐term response trajectories and achievement of CDC with filgotinib were associated with being biologic‐naive and having less severe disease at baseline. Early estimation of sustained and CDC may facilitate patient identification and development of personalized management strategies in UC.

**ClinicalTrials.gov Identifier:**

NCT02914522.

## Graphical abstract

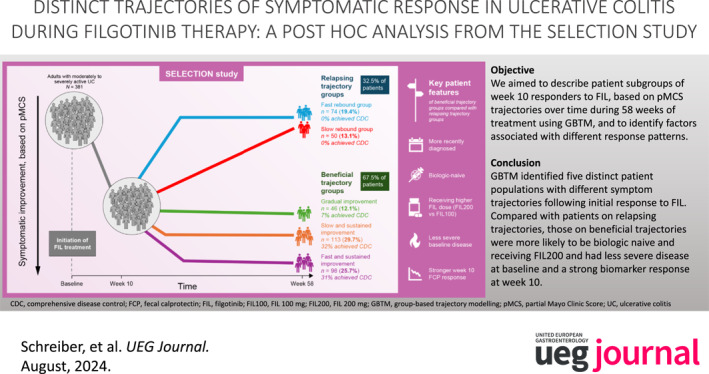

**Key summary**

**Summarize the established knowledge on this subject**

- Filgotinib is approved for the treatment of ulcerative colitis.
- Patient responses to therapy vary, and factors associated with responses are poorly defined.

**What are the significant and/or new findings of this study?**

- We identified distinct patient populations based on symptom trajectories with continued filgotinib treatment.
- Beneficial outcome was associated with being biologic‐naive, receiving filgotinib 200 mg and having less severe disease at baseline.
- This study may enable early estimation of long‐term filgotinib response and aid the development of personalized management strategies.

## INTRODUCTION

Ulcerative colitis is a relapsing and remitting inflammatory bowel disease (IBD) characterized by continuous mucosal inflammation.^1^ Despite the availability of advanced treatments, UC is associated with a substantial disease burden, reduced health‐related quality of life (HRQoL), and the development of complications.^2^, ^3^

Treatments for UC aim to induce and maintain clinical and endoscopic remission.^4^, ^5^ However, individual patient responses to therapies vary,^6^ and factors associated with responses to treatment are not well defined. In clinical trials, efficacy is typically assessed using individual landmark endpoints. Based on these metrics, fewer than half of patients achieve the goal of clinical or endoscopic remission,^7^, ^8^, ^9^, ^10^, ^11^ and responder populations for the different endpoints only partially overlap.^12^ In addition, the disease course varies considerably even in patients who achieve remission, with some patients experiencing rapid benefits and others demonstrating a delayed or gradual response. Furthermore, patients with an initial response to treatment may experience a loss of response over time.^13^, ^14^

Although our clinical experience suggests that a rapid initial clinical response to treatment may be associated with superior long‐term outcomes compared with a slower response, quantitative data to support this notion are lacking. One potential explanation for this circumstance is that landmark clinical endpoints do not provide information regarding dynamic changes in the disease course that are relevant prognostic factors. Therefore, additional information is needed to assess response to treatment, for example, repeated measures of inflammatory biomarker concentrations or clinical symptoms (e.g., partial Mayo Clinic Score [pMCS]) over time, either alone or in combination with landmark endpoints. This approach could enable more sophisticated methods of monitoring early therapeutic responses and predicting long‐term treatment outcomes, thereby assisting the personalization of UC management.

Filgotinib is an oral, once‐daily, Janus kinase 1 preferential inhibitor approved in the European Union, Japan, and the UK for the treatment of adults with moderately to severely active UC with an inadequate response, loss of response, or intolerance to conventional therapy or biological agents.^15^ This approval was based on results from the phase 2b/3 SELECTION study (NCT02914522) in which filgotinib 200 mg was efficacious in inducing and maintaining clinical remission relative to placebo.^8^

Trajectory models can identify groups of individuals with different levels of therapeutic benefit to aid early targeted treatment decisions.^16^ In these *post hoc* analyses of SELECTION data, we describe distinct groups of patients with different symptom trajectories over time during continued filgotinib treatment following response to induction therapy. We evaluated the clinical relevance of grouping patients in this way by assessing the proportions of patients in each group who achieved different landmark endpoints. These included a multi‐component endpoint termed comprehensive disease control (CDC), which encompasses improvements in symptoms, inflammatory biomarkers, HRQoL, and endoscopic activity.^12^ Demographics and clinical characteristics were compared between trajectory groups to identify factors associated with different response trajectories.

## MATERIALS AND METHODS

### Study design and participants

SELECTION (NCT02914522) was a phase 2b/3, double‐blind, placebo‐controlled, randomized trial comprising two induction studies and one maintenance study.^8^ The study protocol was approved by the independent ethics committee or institutional review board at each study site. All participants provided informed consent before participating in the study.^8^

The study population included adults aged 18–75 years with moderately to severely active UC.^8^

Patients were randomized 2:2:1 to receive filgotinib 200 mg, filgotinib 100 mg, or placebo once daily for 11 weeks in Induction Study A (biologic‐naive) and Induction Study B (biologic‐experienced). Responders to filgotinib (defined as patients in clinical remission or with a MCS response at week 10; definitions of these endpoints can be found in the Supplementary Methods) were re‐randomized 2:1 to continue their induction filgotinib dose or to receive placebo in the 47‐week maintenance study (weeks 11–58).

### Group‐based trajectory modeling

GBTM is a technique applied to find distinct clusters of individuals with similar trajectories within an overall population.^17^ It applies finite mixture models that assume the population is composed of a mixture of distinct groups defined by their response trajectories over time. Here, GBTM was employed to model pMCS symptom trajectories over time among patients who responded to filgotinib treatment at week 10. To maximize the separation between the different groups of induction responders, the GBTM included only patients who received filgotinib (either 200 or 100 mg), who entered the maintenance study in SELECTION, and who did not change treatment arms during re‐randomization. Data from patients treated with either dose of filgotinib (i.e. 200 or 100 mg) in SELECTION were pooled for the GBTM analysis.

The model parameters were estimated using an expectation–maximization algorithm, which performed a “maximum likelihood estimation” —a technique that maximizes the chance that the model matches the observed data. The optimal model with respect to the polynomial fit was selected by minimizing the Bayesian information criterion, which further increases the validity of the model by limiting overfitting of the data.^17^ The maximum number of groups in the model was pre‐specified.

Patients were clustered into trajectories based on their pMCS (defined as the sum of the Physician's Global Assessment, rectal bleeding, and stool frequency subscores; ranging from a total of 0–9), which was assessed at baseline and at 12 subsequent time points during the study. For patients who discontinued the study and therefore had missing data, a last observation carried forward (LOCF) imputation approach was used (further details are available in the Supplementary Materials). Baseline patient characteristics and other variables were compared between the groups described by the model using descriptive statistics.

To assess the validity of using pMCS (instead of MCS) over time as a measure of disease activity and to identify the clinical significance of differences between groups, the proportions of patients achieving traditional landmark endpoints were compared between trajectory groups. These endpoints were: clinical remission (defined by use of endoscopic, rectal bleeding, and stool frequency subscores)^8^; corticosteroid (CS)‐free clinical remission; MCS response and remission; pMCS response and remission; endoscopic response and remission; two different thresholds of biological remission (FCP < 150 and < 250 μg/g); and Inflammatory Bowel Disease Questionnaire (IBDQ) remission. The full definitions for all landmark endpoints are provided in the Supplementary Methods. In addition, a patient was considered to have achieved CDC through the simultaneous achievement of pMCS remission, endoscopic response, biological remission (FCP <150 μg/g), and IBDQ remission.^12^ For endpoint analyses, data were reported using non‐responder imputation of missing data for binary variables, and as observed for all other variables. Concordance analysis was conducted to evaluate consistency across the landmark endpoints at week 58.

## RESULTS

### Description of symptom trajectories using GBTM

**FIGURE 1:**
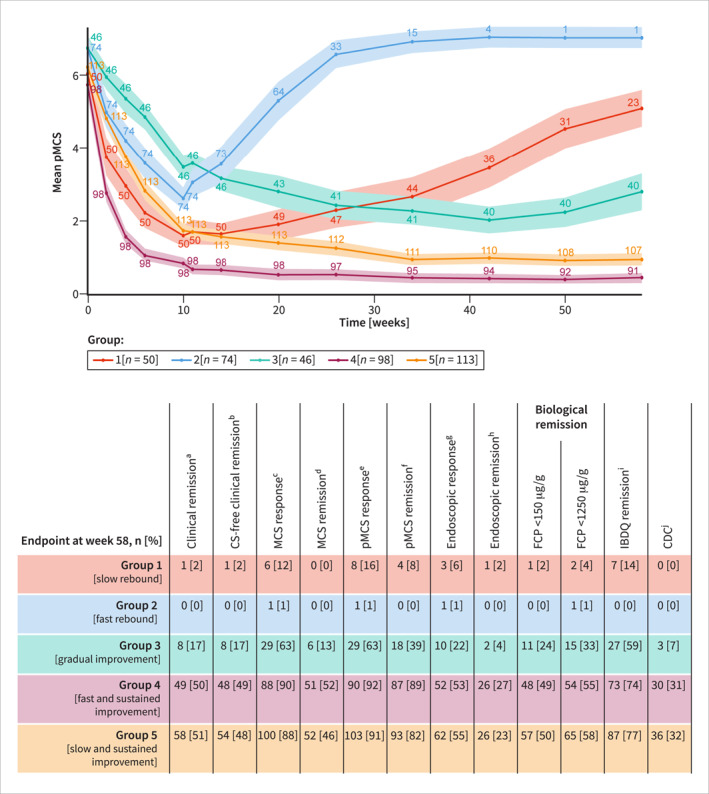
Grouping of pooled filgotinib‐treated patient symptom trajectories using GBTM, and the proportion of patients in each trajectory group achieving landmark endpoints at week 58. Numbers on the graph are numbers of patients with an available pMCS measurement at a specific time point. Shaded regions on the graph represent 95% CIs, and were calculated using the student's *t*‐distribution. pMCS is the sum of Physician's Global Assessment, rectal bleeding, and stool frequency subscores. MCS is the sum of endoscopic, Physician's Global Assessment, rectal bleeding, and stool frequency subscores. Missing data in the GBTM were imputed using LOCF. Missing data in the landmark endpoints were imputed using non‐responder imputation. CDC, comprehensive disease control; CI, confidence interval; CS, corticosteroid; FCP, faecal calprotectin; GBTM, group‐based trajectory modelling; IBDQ, inflammatory bowel disease questionnaire; LOCF, last observation carried forward; MCS, mayo clinic score; pMCS, partial mayo clinic score, UC, ulcerative colitis. ^a^Clinical remission was defined as a Mayo endoscopic subscore of 0 or 1, a rectal bleeding subscore of 0, and a decrease in the stool frequency subscore of at least 1 point from induction baseline to achieve a subscore of 0 or 1. ^b^CS‐free clinical remission was defined as clinical remission with no CS use for the indication of UC for at least 6 months before week 58. ^c^An MCS response was defined as a reduction in the MCS of at least 3 points and at least 30% from induction baseline, with an accompanying decrease in the rectal bleeding subscore of at least 1 point or an absolute rectal bleeding subscore of 0 or 1. ^d^MCS remission was defined as a MCS of 2 of less and no single subscore (endoscopic, Physician’s Global Assessment, rectal bleeding, and stool frequency) greater than 1. ^e^A pMCS response was defined as a decrease in pMCS score of 2 points or more and 30% from induction baseline. ^f^pMCS remission was defined as a pMCS of 2 or less and no single subscore (Physician’s Global Assessment, rectal bleeding, or stool frequency) greater than 1. ^g^An endoscopic response was defined as an endoscopic subscore of 1 or less. ^h^Endoscopic remission was defined as an endoscopic subscore of 0. ^i^IBDQ remission was defined as IBDQ score greater than 170. ^j^CDC was defined as the simultaneous achievement of pMCS remission, an endoscopic response, biological remission (FCP <150 µg/g), and IBDQ remission.

In total, 381 patients treated with filgotinib 200 mg (*n* = 202) or filgotinib 100 mg (*n* = 179) who responded to filgotinib at week 10 and were rerandomized to continue the same dose in the maintenance phase were included in the GBTM analysis. Five groups of different patient responses to therapy were identified based on pMCS measurements from induction baseline to week 58 (Figure 1). The proportions of patients achieving different landmark endpoints in each group were evaluated.

There was clear separation between the five groups, summarized in Box 1. For ease of clinical interpretation, we also considered the trajectory groups in two larger categories based on long‐term patterns in pMCS: those demonstrating relapsing trajectories and those demonstrating beneficial trajectories over 58 weeks. The groups with beneficial patterns in pMCS comprised 67.5% of the patients included in this analysis and consisted of the gradual improvement group (group 3) and the fast and slow sustained improvement groups (groups 4 and 5, respectively).

**BOX 1:**
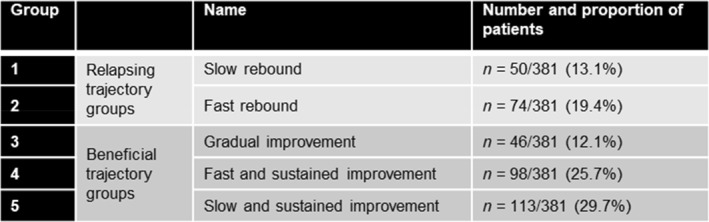
Interpretation of groups identified by GBTM. GBTM, group‐based trajectory modeling.

All groups of patients showed symptom improvement by week 10 (Figure 1), which was expected because the overall patient population was restricted to week 10 responders owing to the re‐randomization design. At this point, the gradual improvement group had the highest mean pMCS, and the lowest mean pMCS was in the fast and sustained improvement group. After week 10, the groups diverged: the fast and sustained improvement group demonstrated maintenance of a reduced mean pMCS; the gradual improvement group and the slow and sustained improvement group continued to experience a reduction of the mean pMCS; and the slow rebound group and the fast rebound group experienced full or partial reversal of the reduction in mean pMCS (Figure 1).

At week 58, the mean pMCS of patients in the relapsing groups had reverted to levels approaching those observed prior to treatment, and no patients achieved CDC (as measured by symptoms, inflammatory biomarkers, endoscopy, and HRQoL at an individual level; Figure 1). Patients in the fast and slow sustained improvement groups maintained their response to treatment, and CDC was achieved in 31% and 32% of patients, respectively. At week 58, the mean pMCS in the gradual improvement group was lower than in the relapsing groups and higher than in the fast and slow sustained improvement groups, and 7% of patients in the gradual improvement group achieved CDC. All other landmark endpoints were achieved by a higher proportion of patients in the beneficial trajectory groups compared with those in the relapsing groups (Figure 1 and described in the Supplementary Results).

The concordance between endpoints is shown in Table 1. Concordance was highest for MCS remission and clinical remission (kappa coefficient: 0.9295; positive predictive value: 98.1%; negative predictive value: 96.6%). The remaining landmark endpoints demonstrated moderate concordance. For pMCS and MCS remissions, the kappa coefficient was 0.5281 (PPV: 54.5%; NPV: 100%). For pMCS remission and clinical remission the kappa coefficient was 0.5532 (PPV: 57.6%; NPV: 99.4%), and for sustained benefit and endoscopic response the kappa coefficient was 0.4755 (PPV: 55.5%; NPV: 96.7%).

**TABLE 1:** Concordance of landmark endpoints at week 58 in patients receiving either 200 or 100 mg filgotinib.

Outcome 1, *n* (%)		Outcome 2, *n* (%)		PPV, % (95% CI)	NPV, % (95% CI)	Kappa coefficient
		Clinical remission^a^				
		Yes (*N* = 115)	No (*N* = 256)			
MCS remission^b^	Yes	106 (92.2)	2 (0.8)	98.1 (93.5, 99.8)	–	0.9295
	No	9 (7.8)	254 (99.2)	–	96.6 (93.6, 98.4)	
		MCS remission^b^				
		Yes (*N* = 108)	No (*N* = 263)			
pMCS remission^c^	Yes	108 (100)	90 (34.2)	54.5 (47.3, 61.6)	–	0.5281
	No	0	173 (65.8)	–	100 (97.9, 100)	
		Clinical remission^a^				
		Yes (*N* = 115)	No (*N* = 256)			
pMCS remission^c^	Yes	114 (99.1)	84 (32.8)	57.6 (50.4, 64.6)	–	0.5532
	No	1 (0.9)	172 (67.2)	–	99.4 (96.8, 100)	
		Endoscopic response^d^				
		Yes (*N* = 127)	No (*N* = 244)			
Sustained benefit^e^	Yes	122 (96.1)	98 (40.2)	55.5 (48.6, 62.1)	–	0.4755
	No	5 (3.9)	146 (59.8)	–	96.7 (92.4, 98.9)	

*Note*: PPV was calculated as the proportion of patients who achieved outcome 2 out of patients achieving outcome 1. NPV was calculated as the proportion of patients who did not achieve outcome 2 out of patients who did not achieve outcome 1. Missing values were imputed using non‐responder imputation. MCS is the sum of endoscopic, Physician's Global Assessment, rectal bleeding, and stool frequency subscores. pMCS is the sum of Physician's Global Assessment, rectal bleeding, and stool frequency subscores.

Abbreviations: CI, confidence interval; MCS, Mayo Clinic Score; NPV, negative predictive value; pMCS, partial Mayo Clinic Score; PPV, positive predictive value.

^a^ Clinical remission was defined as a Mayo endoscopic subscore of 0 or 1, a rectal bleeding subscore of 0, and a decrease in the stool frequency subscore of at least 1 point from the induction baseline to achieve a subscore of 0 or 1.

^b^ MCS remission was defined as a MCS of 2 and no single subscore (endoscopic, Physician's Global Assessment, rectal bleeding, and stool frequency) greater than 1.

^c^ pMCS remission was defined as a pMCS of 2 or less and no single subscore (Physician's Global Assessment, rectal bleeding, or stool frequency) greater than 1.

^d^ An endoscopic response was defined as an endoscopic subscore of 1 or less.

^e^ Sustained benefit was defined as either clinical remission^c^ or an MCS response (defined as a decrease in MCS ≥3 points and ≥30% change from baseline, and a decrease in rectal bleeding subscore of ≥1 point or an absolute rectal bleeding subscore ≤1) at both week 10 and week 58.

### Patient and disease characteristics associated with trajectory groups

Patient characteristics varied between the groups described by the GBTM analysis (Table 2). More patients in the fast and slow sustained improvement groups were diagnosed recently (in the past 3 years) compared with the fast rebound group; 7%–9% of patients in the fast and slow sustained improvement groups had a disease duration of <1 year, and 21%–25% had a disease duration of ≥1 to <3 years, compared with the fast rebound group (5% for <1 year and 16% for ≥1 to <3 years). The fast and slow sustained improvement groups also contained a numerically higher proportion of patients who were treated with filgotinib 200 mg (59%–65%) than the other groups (36%–46%). A higher proportion of patients in the relapsing groups (34% of both groups) were receiving >10 mg/day of systemic corticosteroids compared with the fast and slow sustained improvement groups (20% for both groups). There was also a higher tendency for patients to be biologic‐naive in the fast and sustained improvement group (70%) than in the relapsing groups (35%–58%), particularly when compared with the worst performing group (fast rebound, 35%). The beneficial trajectory groups also had higher proportions of female patients than the relapsing groups (53%–54% vs. 38%–39%).

**TABLE 2:** Baseline demographics and disease characteristics of patients treated with filgotinib 200 mg or filgotinib 100 mg by symptom trajectory group.

Demographic/characteristic	Group 1 (slow rebound) (*n* = 50)	Group 2 (fast rebound) (*n* = 74)	Group 3 (gradual improvement) (*n* = 46)	Group 4 (fast and sustained improvement) (*n* = 98)	Group 5 (slow and sustained improvement) (*n* = 113)
Age, years, median (Q1, Q3)	47 (30, 56)	42 (30, 55)	47 (36, 54)	42 (32, 51)	40 (32, 51)
<65, *n* (%)	48 (96)	68 (92)	46 (100)	94 (96)	106 (94)
≥65, *n* (%)	2 (4.0)	6 (8.1)	0 (0)	4 (4.1)	7 (6.2)
Female sex, *n* (%)	19 (38)	29 (39)	25 (54)	52 (53)	60 (53)
Race					
Asian, *n* (%)	15 (30)	8 (11)	13 (28)	21 (21)	40 (35)
Black, *n* (%)	2 (4)	1 (1)	2 (4)	1 (1)	2 (1)
Not permitted,^a^ *n* (%)	0 (0)	3 (4)	0 (0)	3 (3)	1 (1)
Other, *n* (%)	1 (2)	0 (0)	0 (0)	0 (0)	0 (0)
White, *n* (%)	32 (64)	62 (84)	31 (67)	73 (74)	70 (62)
Ethnicity					
Hispanic or Latino, *n* (%)	2 (4)	2 (3)	2 (4)	2 (2)	4 (4)
Not Hispanic or Latino, *n* (%)	48 (96)	72 (97)	44 (96)	96 (98)	109 (96)
Weight, kg	69 (58, 83)	71 (60, 87)	67 (56, 75)	68 (59, 80)	65 (55, 84)
Height, cm	173 (165, 178)	170 (165, 177)	168 (161, 171)	169 (164, 175)	168 (160, 176)
BMI, kg/m^2^, median (Q1, Q3)	22.8 (20.9, 27.3)	24.4 (20.7, 28.1)	23.7 (20.2, 27.6)	24.0 (20.9, 28.2)	23.4 (20.1, 27.7)
<18.5, *n* (%)	4 (8)	8 (11)	4 (9)	7 (7)	17 (15)
≥18.5 to <25.0, *n* (%)	27 (54)	35 (47)	24 (52)	51 (52)	52 (46)
≥25.0 to <30.0, *n* (%)	15 (30)	19 (26)	12 (26)	30 (31)	23 (20)
≥30, *n* (%)	4 (8)	12 (16)	6 (13)	10 (10)	21 (19)
Time since diagnosis, years, median (Q1, Q3)	5 (2, 9)	7 (4, 13)	7 (3, 13)	8 (2, 14)	6 (2, 12)
<1, *n* (%)	2 (4)	4 (5)	2 (4)	9 (9)	8 (7)
≥1 to <3, *n* (%)	13 (26)	12 (16)	8 (17)	21 (21)	28 (25)
≥3 to <7, *n* (%)	18 (36)	21 (28)	9 (20)	16 (16)	31 (27)
≥7, *n* (%)	17 (34)	37 (50)	27 (59)	51 (52)	46 (41)
Induction study assignment					
Induction Study A, *n* (%)	28 (56)	26 (35)	26 (57)	69 (70)	67 (59)
Induction Study B, *n* (%)	22 (44)	48 (65)	20 (43)	29 (30)	46 (41)
Filgotinib study arm					
100 mg → 100 mg, *n* (%)	27 (54)	47 (64)	26 (57)	40 (41)	39 (35)
200 mg → 200 mg, *n* (%)	23 (46)	27 (36)	20 (43)	58 (59)	74 (65)
Current aminosalicylate use, *n* (%)	42 (84)	44 (59)	35 (76)	78 (80)	93 (82)
Prior vedolizumab use, *n* (%)	9 (18)	28 (38)	11 (24)	11 (11)	22 (19)
Prior vedolizumab failure, *n* (%)	8 (16)	21 (28)	11 (24)	11 (11)	17 (15)
Prior TNF antagonist use, *n* (%)	21 (42)	45 (61)	20 (43)	26 (27)	40 (35)
Prior TNF antagonist failure, *n* (%)	19 (38)	38 (51)	19 (41)	25 (26)	37 (33)
Prior immunomodulator use, *n* (%)	29 (58)	47 (64)	23 (50)	49 (50)	65 (58)
Concomitant systemic corticosteroid dose, mg/day, median (Q1, Q3)	20 (10, 20)	20 (10, 20)	20 (10, 22)	20 (10, 30)	15 (10, 20)
>0 to ≤10, *n* (%)	8 (16)	17 (23)	6 (13)	13 (13)	15 (13)
>10 to ≤20, *n* (%)	11 (22)	16 (22)	8 (17)	11 (11)	14 (12)
>20, *n* (%)	6 (12)	9 (12)	5 (11)	9 (9)	9 (8)
Number of biologics used previously					
0, *n* (%)	29 (58)	26 (35)	25 (54)	69 (70)	67 (59)
1, *n* (%)	8 (16)	15 (20)	9 (20)	15 (15)	21 (19)
2, *n* (%)	5 (10)	17 (23)	9 (20)	8 (8)	14 (21)
≥3, *n* (%)	8 (16)	16 (22)	3 (7)	6 (6)	11 (9.7)
MCS, median (Q1, Q3)	6.00 (5.00, 7.00)	7.00 (6.00, 7.75)	7.00 (6.00, 7.00)	6.00 (5.00, 7.00)	6.00 (5.00, 7.00)
Endoscopic subscore					
2, *n* (%)	18 (36)	22 (30)	7 (15)	47 (48)	49 (43)
3, *n* (%)	32 (64)	52 (70)	39 (85)	51 (52)	64 (57)
Physician's global assessment subscore					
1, *n* (%)	1 (2)	0 (0)	0 (0)	1 (1)	2 (2)
2, *n* (%)	39 (78)	48 (65)	31 (67)	80 (82)	80 (71)
3, *n* (%)	10 (20)	26 (35)	15 (33)	17 (17)	31 (27)
Rectal bleeding subscore					
0, *n* (%)	3 (6)	2 (3)	3 (7)	3 (3)	3 (3)
1, *n* (%)	15 (31)	23 (32)	12 (26)	41 (43)	41 (37)
2, *n* (%)	25 (52)	36 (49)	27 (59)	48 (50)	63 (57)
3, *n* (%)	5 (10)	12 (16)	4 (9)	4 (4)	4 (4)
Stool frequency subscore					
≤1, *n* (%)	7 (14)	4 (5)	0 (0)	15 (15)	16 (14)
2, *n* (%)	21 (42)	24 (32)	14 (30)	54 (55)	43 (38)
3, *n* (%)	22 (44)	46 (62)	32 (70)	29 (30)	54 (48)
FCP concentration, μg/g, median (Q1, Q3)	1232 (305, 2211)	1460 (526, 3072)	1120 (254, 2468)	1285 (579, 2794)	1526 (470, 2678)
<150, *n* (%)	2 (4)	4 (6)	6 (13)	5 (5)	13 (12)
≥150, *n* (%)	48 (96)	68 (94)	40 (87)	91 (95)	97 (88)
≤250, *n* (%)	8 (16)	12 (16)	12 (26)	11 (11)	17 (15)
>250, *n* (%)	42 (84)	60 (81)	34 (74)	85 (87)	93 (82)
CRP concentration, mg/L, median (Q1, Q3)	2 (1, 7)	5 (2, 14)	6 (2, 12)	2 (1, 6)	3 (1, 9)
≤3, *n* (%)	26 (52)	27 (36)	19 (41)	54 (55)	55 (49)
>3, *n* (%)	24 (48)	47 (64)	27 (59)	44 (45)	58 (51)
Lactoferrin concentration, μg/g, median (Q1, Q3)	156 (48, 344)	126 (45, 277)	102 (23, 275)	129 (50, 313)	121 (37, 291)
IBDQ score, median (Q1, Q3)	132 (106, 151)	108 (84, 134)	117 (90, 135)	125 (96, 144)	114 (89, 134)
SF‐36 physical component summary, median (Q1, Q3)	44 (40, 49)	41 (38, 45)	42 (34, 47)	42 (38, 47)	42 (37, 47)
EQ VAS score, median (Q1, Q3)	60 (45, 70)	47 (31, 60)	50 (36, 70)	51 (34, 66)	50 (39, 68)

*Note*: Baseline measures correspond to the last assessment before the first intake. MCS is the sum of endoscopic, Physician's Global Assessment, rectal bleeding, and stool frequency subscores.

Abbreviations: BMI, body mass index; CRP, C‐reactive protein; FCP, faecal calprotectin; IBDQ, inflammatory bowel disease questionnaire; MCS, mayo clinic score; Q1, lower quartile; Q3, upper quartile; SF‐36, 36‐item short form survey; TNF, tumor necrosis factor.

^a^ Local regulators did not allow the collection of race or ethnicity information.

Baseline measures of disease activity also varied between groups. The fast and slow sustained improvement groups generally had a lower baseline endoscopic subscore than the other groups, with numerically higher proportions of patients having a baseline endoscopic subscore of 2 (the lowest recorded as no patient had a baseline endoscopic score of 0 or 1; 43%–48% vs. 15%–36%). There were no clear patterns in baseline IBD‐specific HRQoL (as measured by IBDQ score), or FCP, C‐reactive protein, or lactoferrin concentration across the different groups.

Differences were evident in week 10 assessments between the trajectory groups (Table 3). At week 10, the fast and slow sustained improvement groups had a higher proportion of patients with an FCP concentration of <150 μg/g (42%–59%) and a greater proportion of patients with a reduction in FCP concentration of >50% from baseline (61%–83%) compared with the relapsing groups (FCP <150 μg/g: 22%–30%; >50% decrease in FCP concentration: 50%–54%). In addition, at week 10, a higher proportion of patients in the best performing group (fast and sustained improvement) than the worst performing group (fast rebound) achieved an endoscopic response (53% vs. 24%), a rectal bleeding subscore of 0 (99% vs. 74%) and a stool frequency subscore of 0 (69% vs. 24%).

**TABLE 3:** Week 10 assessment of patients treated with filgotinib 200 mg or filgotinib 100 mg by symptom trajectory group.

Characteristic	Group 1 (slow rebound) (*n* = 50)	Group 2 (fast rebound) (*n* = 74)	Group 3 (gradual improvement) (*n* = 46)	Group 4 (fast and sustained improvement) (*n* = 98)	Group 5 (slow and sustained improvement) (*n* = 113)
pMCS remission^a^, *n* (%)	37 (74)	28 (38)	7 (15)	92 (94)	83 (73)
Endoscopic response^b^, *n* (%)	23 (46)	18 (24)	12 (26)	52 (53)	40 (35)
Physician's global assessment subscore					
0, *n* (%)	17 (35)	12 (16)	0	55 (56)	27 (24)
1, *n* (%)	27 (56)	38 (51)	24 (55)	40 (41)	69 (62)
2, *n* (%)	4 (8)	22 (30)	18 (41)	3 (3)	16 (14)
3, *n* (%)	0	2 (3)	2 (5)	0	0
Missing, *n*	2	0	2	0	1
Rectal bleeding subscore					
0, *n* (%)	43 (90)	55 (74)	30 (68)	97 (99)	99 (88)
1, *n* (%)	5 (10)	19 (26)	13 (30)	1 (1)	13 (12)
2, *n* (%)	0	0	1 (2)	0	0
Missing, *n*	2	0	2	0	1
Stool frequency subscore					
0, *n* (%)	20 (42)	18 (24)	3 (7)	68 (69)	47 (42)
1, *n* (%)	23 (48)	33 (45)	23 (52)	27 (28)	53 (47)
2, *n* (%)	5 (10)	17 (23)	14 (32)	3 (3)	12 (11)
3, *n* (%)	0	6 (8)	4 (9)	0	0
Missing, *n*	2	0	2	0	1
FCP <150 μg/g, *n* (%)	15 (30)	16 (22)	14 (30)	58 (59)	48 (42)
>50% decrease in FCP, *n* (%)	27 (54)	37 (50)	30 (65)	81 (83)	69 (61)
>50% decrease in CRP, *n* (%)	25 (50)	47 (64)	29 (63)	52 (53)	66 (58)
IBDQ score, median (Q1, Q3)	184 (171, 199)	176 (146, 196)	170 (150, 182)	188 (168, 205)	184 (153, 198)
Missing, *n*	2	0	2	0	1
IBDQ remission^c^, *n* (%)	36 (72)	43 (58)	18 (39)	71 (72)	68 (60)
Geboes histological remission^d^, *n* (%)	20 (40)	15 (20)	12 (26)	45 (46)	38 (34)
SF‐36 physical component summary, median (Q1, Q3)	53 (46, 56)	50 (43, 54)	47 (43, 54)	52 (46, 55)	52 (46, 55)
Missing, *n*	2	0	2	0	1
EQ VAS score, median (Q1, Q3)	79 (70, 90)	74 (60, 81)	71 (60, 81)	80 (70, 89)	76 (61, 86)
Missing, *n*	2	0	2	0	1

*Note*: pMCS is the sum of Physician's Global Assessment, rectal bleeding, and stool frequency subscores. For binary variables, missing data were imputed using non‐responder imputation. For all other variables, data are presented as observed, with the number of missing values indicated.

Abbreviations: CRP, C‐reactive protein; FCP, faecal calprotectin; IBDQ, inflammatory bowel disease questionnaire; pMCS, partial mayo clinic score; Q1, lower quartile; Q3, upper quartile; SF‐36, 36‐item short form survey.

^a^ pMCS remission was defined as a pMCS of 2 or less and no single subscore (Physician's Global Assessment, rectal bleeding, or stool frequency) greater than 1.

^b^ An endoscopic response was defined as an endoscopic subscore of 1 or less.

^c^ IBDQ remission was defined as an IBDQ score greater than 170.

^d^ Geboes histological remission was defined as a grade 0 score of 0.3 or less, a grade 1 score of 1.1 or less, a grade 2a score of 2A.3 or less, a grade 2b score of 2B.0, a grade 3 score of 3.0, a grade 4 score of 4.0, and a grade 5 score of 5.0.

## DISCUSSION

Through trajectory modeling, we have described distinct subpopulations of patients with unique pMCS‐based trajectories, among those with moderately to severely active UC who responded to filgotinib induction therapy and continued treatment up to week 58 in the SELECTION study. Trajectories ranged from a fast and sustained response, to gradual improvement, to relapse. The patient subpopulations had differences in baseline characteristics and in the likelihood of achieving CDC at week 58. High proportions of patients in the fast and slow sustained improvement groups achieved CDC and this finding was reflected in the achievement of different landmark outcomes.

Because all patients included in our analysis were responders at week 10, owing to the re‐randomization design of SELECTION, all groups of patients had an acute therapeutic benefit from filgotinib, with a reduced mean pMCS reported in all subpopulations in the first 10 weeks of treatment. Nevertheless, differences between trajectory groups in clinical characteristics and achievement of landmark endpoints at week 10 suggested some early separation in symptom load between groups.

After week 10, symptom trajectories further diverged. Compared with the relapsing groups, the beneficial long‐term trajectory groups, which included 67.5% of all patients, comprised a higher proportion of patients who achieved the traditional landmark outcomes at week 58. Given that the landmark outcomes demonstrated only moderate levels of concordance among them, this finding further validates the use of the pMCS over time as a clinically significant measure of disease activity.

Patients with trajectories of sustained improvements in symptoms at week 58 were more likely to have been diagnosed in the past 3 years, be receiving filgotinib 200 mg, and be biologic‐naive compared with patients who were following relapsing trajectories. Furthermore, patients with beneficial trajectories were more likely to have a low baseline endoscopic subscore and a strong FCP response at week 10 compared with patients who were in relapsing trajectories. Overall, our results suggest that patients with beneficial trajectories tended to have less refractory UC than those with relapsing trajectories. The association of demographic and clinical factors with pMCS over time is being further evaluated using a mixed model for repeated measures and findings will be reported in a separate publication.

In the beneficial symptom trajectory groups, a higher proportion of patients achieved FCP <150 μg/g at week 10 compared with the relapsing groups (30%–59% vs. 22%–30%). These patterns were maintained for the duration of the study, with a numerically higher proportion of patients in the sustained improvement groups reaching FCP <150 μg/g than in the other groups at week 58 (49%–50% vs. 0%–24%). Of particular interest was that at week 10 in the most desirable symptom trajectory group (fast and sustained improvement), 59% of patients had an FCP concentration of <150 μg/g (vs. 22%–42% for other groups), and 83% demonstrated a reduction in FCP concentration of >50% relative to baseline (vs. 50%–65% for other groups). These findings raised the possibility that FCP concentration could be an early, non‐invasive, prognostic marker of the long‐term pMCS‐based trajectory that a patient may follow. Further analysis of FCP concentration as a predictive marker is forthcoming.

In addition to the traditional landmark endpoints, we also assessed the likelihood of patients in each trajectory group achieving a combined endpoint termed CDC, which comprises symptomatic (pMCS) remission, endoscopic response, inflammatory biomarker control, and IBD‐specific quality of life (IBDQ) remission.^12^ As the achievement of CDC requires multiple criteria to be met, it is a stringent endpoint, and achievement represents broad and far‐reaching disease control at the individual patient level. Here, the groups demonstrating fast and slow sustained improvements in symptoms comprised a substantially higher proportion of patients achieving CDC at week 58 than the remaining groups (31%–32% vs. 0%–7%, respectively). Considering the differential early FCP concentrations between trajectory groups, this raises the possibility of improved prediction of long‐term comprehensive disease management, spanning improvements to symptoms, HRQoL, and mucosal healing, after just a few months of treatment.

It is evident in the literature that the medical management of UC is shifting towards personalized medicine. GBTM has been used to study trajectories in response to ozanimod in patients with UC^18^, ^19^, ^20^, ^21^ and infliximab in patients with Crohn's disease.^22^ The results of GBTM graphically illustrate the heterogeneity of response in a patient population, and demonstrate the importance of patient‐specific monitoring of disease activity, particularly at early timepoints, for predicting long‐term treatment outcomes. In addition, GBTM can be combined with the evaluation of factors that are associated with treatment success. Herein, our description of the characteristics of patients who tend to follow beneficial trajectories would be valuable for clinicians in developing personalized medical management strategies by considering patients' demographics and clinical characteristics in patients receiving filgotinib treatment.

Our study had several strengths. The unique application of GBTM to data that were collected within the rigorous setting of a large, phase 2b/3 clinical trial allowed qualitatively distinct patient subpopulations to be identified, and distinguished real differences between individuals from chance variation. In contrast to traditional statistical approaches that require human input to select variables for evaluation, GBTM is an unsupervised technique^23^ that, in our work, allowed the classification of patients according to different pMCS‐driven patterns. In this way, GBTM produces more robust results than traditional statistics. An overview of the GBTM methodology has been provided by Nagin et al.^24^ Although the methodology of GBTM is more complicated than traditional statistical methods, it produces a visual and accessible output for both technical and non‐technical audiences. Together with our work, trajectory modeling for other therapies may allow further identification of distinct subpopulations in UC based on initial response, disease pathophysiology, and baseline disease characteristics.^25^, ^26^, ^27^ Such further analyses are forthcoming,^28^, ^29^ also highlighting the potential of applying GBTM to UC disease pathophysiology under different therapies.

We performed GBTM using pMCS, instead of the two‐item patient‐reported outcome (PRO2) score. PRO2 is made up of the two patient‐reported subscores of pMCS (rectal bleeding and stool frequency) and is an easy, non‐invasive measure that is often used in the assessment of UC disease activity.^30^ PRO2 does not include the Physician's Global Assessment subscore, and thus, it is a less subjective scoring instrument than pMCS. However, including the Physician's Global Assessment subscore in pMCS enables results to reflect clinical practice, where the physician's evaluation of patients' outcomes is a key component in decision making. Furthermore, pMCS in this study was largely determined by the rectal bleeding and stool frequency subscores; therefore, we speculate that the results would not have been substantially different if PRO2 had been used instead of pMCS to model response trajectories. Indeed, a high concordance is observed between pMCS remission and clinical remission (which includes endoscopic, rectal bleeding and stool frequency subscores; all elements of MCS, except for the Physician's Global Assessment subscore). Another alternative to pMCS is the modified MCS, which is made up of rectal bleeding, stool frequency and endoscopic subscores. Employing a measure that includes endoscopic activity could potentially reduce the subjectivity of measures and provide a more intrinsic assessment of disease activity. Importantly, GBTM requires the input of multiple measures over time for each patient. Endoscopic data were collected at infrequent timepoints in the SELECTION trial, and therefore, the use of modified MCS in the GBTM would result in multiple missing data points. To compensate for this limitation, we have assessed the concordance of clinical remission (which uses the same subscores as the modified MCS) and endoscopic response with pMCS remission.

A further limitation of these analyses is that the population included only week 10 filgotinib responders owing to the re‐randomization design of SELECTION. The study design also led to a relatively small number of patients in the trajectory groups, especially for the groups that were associated with less desirable symptom trajectories over time. A treat‐through design may have been more suitable for this type of analysis. In addition, the potential effect of imputation of missing data must be considered. Although the choice to use LOCF was an informed one using a meaningful and clinically feasible assumption, the true symptom trajectory for patients who discontinued the study is unknown. Furthermore, there is little evidence on the anticipated impact of covariance misspecification in GBTM,^31^ and it was not possible to investigate the potential impact that this may have had on our analyses. Nonetheless, the use of polynomial fit may, in part, mitigate the effect of misspecifications on model performance.

In conclusion, we described unique subpopulations of patients with UC based on their response to filgotinib over time. Combining early data on symptomatic (pMCS) response and associated variables could be a valuable tool for predicting the long‐term symptom trajectory and week 58 individual benefits for patients receiving filgotinib. This could improve the overall management of UC, allowing for a personalized monitoring plan that can be adjusted based on the anticipated clinical trajectory. Future work should use treat‐through designs to explore symptom trajectories in different cohorts (including in responder subgroups) and compare different therapeutic classes in relation to other outcomes and specific factors (e.g., innovative biomarkers and histology data). This would enable a more sophisticated prediction of response and choice of therapy to optimize the chances of patients with UC achieving CDC.

## AUTHOR CONTRIBUTIONS

Guarantor of the article: Stefan Schreiber. All authors were involved in the conception and/or methodological design of the study. Toshifumi Hibi, Stefan Schreiber, Louis Dron, and Corinne Jamoul were involved in data acquisition and analysis, and Louis Dron performed statistical analysis. All authors were involved in data interpretation. All authors contributed to the development of the article and approved the final version.

## CONFLICT OF INTEREST STATEMENT

SS reports personal fees from AbbVie, Amgen, Arena Pharmaceuticals, Biogen, Bristol Myers Squibb, Celgene, Celltrion, Dr Falk Pharma, Eli Lilly, Ferring Pharmaceuticals, Fresenius Kabi, Galapagos/Gilead Sciences, Hikma Pharmaceuticals, I‐Mab, Janssen Pharmaceuticals, Morphic Therapeutic, MSD, Mylan, Pfizer, Protagonist Therapeutics, Provention Bio, Sandoz/Hexal, Takeda, Theravance Biopharma, and Ventyx Biosciences. BGF reports grants and personal fees from AbbVie, Amgen, AstraZeneca, Bristol Myers Squibb, Janssen Biotech/Centocor, Johnson & Johnson/Janssen, Pfizer, Receptos, and Takeda; and personal fees from Ablynx, ActoGeniX, AdMIRx, Akebia Therapeutics, Allergan, Atlantic Pharmaceuticals, Avaxia Biologics, Avir Pharma, Baxter Healthcare Corporation, Biogen, BiomX Israel, Biora Therapeutics (formerly Progenity), Boehringer Ingelheim, Boston Pharmaceuticals, Calypso Biotech, Celgene, Elan/Biogen, Eli Lilly, enGene, Ferring Pharmaceuticals, Galapagos, Genentech/Roche, gIcare Pharma, Gilead Sciences, Given Imaging, Gossamer Bio, GSK, Inception IBD, Ironwood Pharmaceuticals, Japan Tobacco Company, Kyowa Hakko Kirin, Lexicon Pharmaceuticals, Lycera, Mesoblast, MSD, Nestlé, Nextbiotix, Novartis, Novo Nordisk, ParImmune, Prometheus Therapeutics and Diagnostics, Protagonist Therapeutics, Qu Biologics, Salix Pharmaceuticals, Shire, Sienna Biopharmaceuticals, Sigmoid Pharma, Synergy Pharma, Takeda Oncology (formely Millennium Pharmaceuticals), Teva Pharmaceuticals, TiGenix, Tillotts Pharma, UCB, Vertex Pharmaceuticals, VHsquared, Vivelix Pharmaceuticals, Wyeth, Zealand Pharma, and Zyngenia. EL has received educational and research grants from AbbVie, Fresenius Kabi, Janssen Pharmaceuticals, Pfizer, and Takeda; speaker fees from AbbVie, Celgene, Dr Falk Pharma, Ferring Pharmaceuticals, Galapagos, Janssen Pharmaceuticals, Pfizer, and Takeda; advisory board fees from AbbVie, Arena Pharmaceuticals, Bristol Myers Squibb, Celgene, Eli Lilly, Ferring Pharmaceuticals, Gilead Sciences/Galapagos, Janssen Pharmaceuticals, MSD, Pfizer, and Takeda; and consulting fees from AbbVie. TaH has received a joint research agreement with Kissei Pharmaceutical and EA Pharma; grants from AbbVie, Daiichi Sankyo, EA Pharma, JIMRO, Kyorin Pharmaceutical, Mitsubishi Tanabe Pharma Corporation, Mochida Pharmaceutical, Nippon Kayaku, Pfizer, Takeda, and Zeria Pharmaceutical; and consulting and lecture fees from AbbVie, Bristol Myers Squibb, EA Pharma, Eli Lilly, Gilead Sciences, Janssen Pharmaceuticals, JIMRO, Kyorin Pharmaceutical, Mitsubishi Tanabe Pharma Corporation, Mochida Pharmaceutical, Pfizer, and Takeda. ToH has received lecture fees from Abbvie, Janssen Pharmaceuticals, JIMRO, Mitsubishi Tanabe Pharma Corporation, Mochida Pharmaceutical, Pfizer, Sandoz, Takeda, and Zeria Pharmaceutical; advisory/consulting fees from Abbvie, Celltrion, EA Pharma, Eli Lilly, Gilead Sciences, Mitsubishi Tanabe Pharma Corporation, Takeda, and Zeria Pharmaceutical; research grants from AbbVie, Activaid, Alfresa Pharma Corporation, Bristol Myers Squibb, Eli Lilly Japan, Ferring Pharmaceuticals, Gilead Sciences, Janssen Pharmaceutical, JMDC, Mochida Pharmaceutical, Nippon Kayaku, Pfizer Japan, and Takeda; and study group sponsorship from AbbVie, Alfresa Pharma Corporation, JIMRO, Kyorin Pharmaceutical, Miyarisan Pharmaceutical, Mochida Pharmaceutical, and Zeria Pharmaceutical. LD was an employee of Cytel at the time of this study, which received funding from Galapagos NV to conduct the analyses. CJ was a contractor at Galapagos at the time of this study. KH and AO were employees and shareholders of Galapagos at the time of this work and are now employees of Alfasigma S.p.A. HP and VT were employees and shareholders of Galapagos at the time of this study. LP‐B has fees from AbbVie, Abivax, Adacyte Therapeutics, Alimentiv, Alma Bio Therapeutics, Amgen, Applied Molecular Transport, Arena Pharmaceuticals, Biogen, Bristol Myers Squibb, Celltrion, CONNECT Biopharma, Cytoki Pharma, Eli Lilly, Enthera, Ferring Pharmaceuticals, Fresenius Kabi, Galapagos, Genentech, Gilead Sciences, Gossamer Bio, GSK, H.A.C. Pharma, IAG Image Analysis Group, Index Pharmaceuticals, Inotrem, Janssen Pharmaceuticals, Medac, Mopac, Morphic Therapeutic, MSD, Nordic Pharma, Norgine, Novartis, OM Pharma, Ono Pharmaceutical, OSE Immunotherapeutics, Pandion Therapeutics, Par’Immune, Pfizer, Prometheus, Protagonist Therapeutics, Roche, Roivant, Samsung, Sandoz, Sanofi, Takeda, Theravance Biopharma, Thermo Fisher Scientific, TiGenix, Tillotts Pharma, VectivBio, Ventyx Biosciences, Viatris, Vifor Pharma, and YSOPIA Bioscience.

## Supporting information

Supporting Information S1

## Data Availability

Anonymized individual patient data will be shared upon request for research purposes dependent upon the nature of the request, the merit of the proposed research, and the availability of the data and their intended use. The full data sharing policies for Gilead Sciences, Inc., and Galapagos NV can be found at https://www.gileadclinicaltrials.com/en/transparency‐policy and https://www.clinicaltrials‐glpg.com/us/en/data‐transparency.html, respectively.
