# Supplementary material for: Distinct trajectories of symptomatic response in ulcerative colitis during filgotinib therapy: A post hoc analysis from the SELECTION study
Source: United European Gastroenterol J. 2024 Oct 25;12(9):1243–55. doi: 10.1002/ueg2.12686 (PMC11578847; doi:10.1002/ueg2.12686)
Supplement: Supplementary file 1 — Supporting Information S1 [file UEG2-12-1243-s001.docx]

**Distinct trajectories of symptomatic response in ulcerative colitis during filgotinib therapy: a post hoc analysis from the SELECTION study**
Stefan Schreiber,^a^ Brian G. Feagan,^a^ Edouard Louis, Tadakazu Hisamatsu, Toshifumi Hibi, Louis Dron, Corinne Jamoul, Haridarshan Patel, Kristina Harris, Virginia Taliadouros, Alessandra Oortwijn, and Laurent Peyrin-Biroulet

^a^Shared first authorship.

**PLAIN LANGUAGE SUMMARY**

Ulcerative colitis (UC) is a long-term condition in which parts of the colon and rectum become inflamed. Multiple treatments are available, but these do not work for everyone. Early prediction of whether a particular treatment will benefit patients who continue to take it could allow for more quick and effective achievement of symptom-free disease control.

Here, we analyzed data from SELECTION, a trial that investigated the safety and efficacy of filgotinib in patients with UC for 58 weeks. We describe patients in 5 distinct groups based on how their symptom severity changed over time, each with varying levels of therapeutic benefit. We then compared patient characteristics between the groups and identified factors linked to different response levels.

Among the cohort of patients, we found that better responses to treatment were seen in patients who were female, who had not taken a particular class of medicines before (called ‘biologics’), and who had less severe disease before starting filgotinib treatment.

Early prediction of long-term responses to treatment can help in the management of patients with UC by enabling healthcare professionals to make more informed treatment decisions. We will report further data on potential factors that may influence and/or predict the response to treatment in a future publication.

**SUPPLEMENTARY METHODS**

Last observation carried forward (LOCF) imputation

The LOCF imputation method was selected based on initial analyses of the data. During the maintenance study, the most common reason for patient discontinuation was disease worsening.^1^ Therefore, employment of LOCF enabled the most accurate classification of patients into different trajectory groups by ensuring the symptom trajectory continued to reflect the disease severity observed at the time of study discontinuation. Missing data at week 58 were not imputed with data collected any earlier than week 26. Based on prior experience, the model was restricted to a maximum of 5 groups to allow for sufficient representation of the data while facilitating interpretation.

Definitions of Landmark Endpoints

Clinical remission was defined as a Mayo endoscopic subscore of 0 or 1, a rectal bleeding subscore of 0, and a decrease in the stool frequency subscore of at least 1 point from induction baseline to achieve a subscore of 0 or 1. Corticosteroid (CS)-free clinical remission was defined as clinical remission with no CS use for the indication of UC for at least 6 months before week 58. A Mayo Clinic Score (MCS) response was defined as a reduction in the MCS of at least 3 points and at least 30% from induction baseline, with an accompanying decrease in the rectal bleeding subscore of at least 1 point or an absolute rectal bleeding subscore of 0 or 1. MCS remission was defined as a MCS of 2 of less and no single subscore (endoscopic, Physician’s Global Assessment, rectal bleeding, and stool frequency) greater than 1. A partial Mayo Clinic Score (pMCS) response was defined as a decrease in pMCS score of 2 points or more and 30% from induction baseline. pMCS remission was defined as a pMCS of 2 or less and no single subscore (Physician’s Global Assessment, rectal bleeding, or stool frequency) greater than 1. An endoscopic response was defined as an endoscopic subscore of 1 or less. Endoscopic remission was defined as an endoscopic subscore of 0. Biological remission was defined using 2 thresholds: fecal calprotectin (FCP) <150 µg/g or 250 µg/g. Inflammatory Bowel Disease Questionnaire (IBDQ) remission was defined as IBDQ score greater than 170. Comprehensive disease control (CDC) was defined as the simultaneous achievement of pMCS remission, an endoscopic response, biological remission (FCP <150 µg/g), and IBDQ remission. Geboes histological remission was defined as a grade 0 score of ≤0.3, grade 1 of ≤1.1, a grade 2a score of ≤2A.3, a grade 2b score of 2B.0, a grade 3 score of 3.0, a grade 4 score of 4.0, and a grade 5 score of 5.0.

**SUPPLEMENTARY RESULTS**

Achievement of Landmark Endpoints and CDC at Week 58

In the sustained improvement groups, a higher proportion of patients achieved clinical remission (50%–51%), CS-free clinical remission (48%–49%), MCS response (88%–90%), MCS remission (46%–52%), pMCS response (91%–92%), pMCS remission (82%–89%), endoscopic response (53%–55%), and endoscopic remission (23%–27%) at week 58 than the relapsing groups (0%–2%, 0%–2%, 1%–12%, 0%, 1%–16%, 0%–8%, 1%–6%, and 0%–2%, respectively). The proportion of patients achieving each landmark endpoint in the gradual improvement group was between that of the sustained improvement groups and the relapsing groups (17%, 17%, 63%, 13%, 63%, 39%, 22%, and 4%, respectively). In general, a higher proportion of patients in the beneficial trajectory groups achieved biological remission than those in the other groups (24%–58% versus 0%–4%). Similarly, in the sustained improvement groups, 31%–32% of patients achieved CDC at week 58. This was higher than in the gradual improvement group (7%), and no patients achieved CDC in the relapsing groups.

**SUPPLEMENTARY REFERENCES**

1. Feagan BG, Danese S, Loftus EV, Jr., Vermeire S, Schreiber S, Ritter T, et al. Filgotinib as induction and maintenance therapy for ulcerative colitis (SELECTION): a phase 2b/3 double-blind, randomised, placebo-controlled trial. Lancet. 2021;397:2372–2384. 10.1016/s0140-6736(21)00666-8.
